# Homoisoflavonoids and the Antioxidant Activity of *Ophiopogon japonicus* Root

**Published:** 2017

**Authors:** Yancui Wang, Feng Liu, Zongsuo Liang, Liang Peng, Bangqing Wang, Jing Yu, Yingying Su, Cunde Ma

**Affiliations:** a *College of Life Sciences, Northwest A & F University, Yangling, Shaanxi 712100, China.*; b *School of Pharmacy, Health Science Center, Xi›an Jiaotong University, Xi’an, Shaanxi Province, China.*; c *Shaanxi institute of international trade & commerce, Xiangyang 712046, China. *; d *Shaanxi Buchang Pharmaceutical Co, Ltd, Shaanxi, China.*

**Keywords:** *Ophiopogon japonicas*, Homoisoflavonoids, LCMS/MS analysis, Antioxidant activity

## Abstract

The root of *Ophiopogon japonicus* has been used as a traditional Chinese medicine and also a functional food ingredient for a long time in China. In the present study, 17 different homoisoflavonoid compounds were identified in the root extract of* O. japonicus* by HPLC–DAD and LCMS/MS analyses. The antioxidant activity of the of chloroform/methanol (1:1, v/v), methanol and 70% ethanol extracts, and two major isolated homoisoflavonoid compounds (methylophiopogonanone A and methylophiopogonanone B) from *O. japonicus* root were investigated by various *in-vitro* assays. Methylophiopogonanone B showed the highest antioxidant ability according to four antioxidant methods. Among the extracts, the chloroform/methanol extract which contained high amounts of homoisoflavonoids was found to exhibit the strongest antioxidant activity. The results showed that *O. japonicus* root can be regarded as a potential source of homoisoflavonoids and natural antioxidant.

## Introduction

The genus *Ophiopogon* (Liliaceae) comprises approximately fifty species and some varieties, and are widespread throughout Southeast Asia. Thirty-three *Ophiopogon* species and four varieties were found in China (1). *Ophiopogon japonicus* Ker-Gawl (Liliaceae) is widely distributed in Sichuan and Zhejiang provinces of China. The root of *O. japonicus* has high medicinal and edible value. As a traditional Chinese medicine, it is considered effective in treating diseases such as thrombosis, myocardial ischemia, arrhythmias, respiratory disease, and hyperglycemia (2–4). 

Homoisoﬂavonoids belong to a type of special flavonoids. Their B- and C-rings were connected by an additional CH_2_ group (5). Homoisoflavonoids have been reported to be responsible for excellent biological activities such as anti-inflammatory (6) and anti-hyperglycemia (3). Homoisoflavonoids also have been detected as potent phosphodiesterase inhibitors (7). Previous studies have revealed that *O. japonicus* is abundant in homoisoflavonoids (8–10).

Antioxidants have been widely used in medicines and foods. They can be of synthetic or natural origin. However, some side effects of synthetic antioxidants have been reported (11). Therefore, there is an increasing interest in researching on natural compounds with antioxidant properties in recent years. In this context, many plants, particularly their flavonoids and phenolic compounds were evaluated for their antioxidant activities (12–15).

In previous investigations, a multitude of homoisoflavonoids have been isolated from *O. japonicus* root. Moreover, the antioxidant activity of polysaccharides of *O. japonicus* root was earlier reported (16, 17). However, there is no report about the antioxidant activity of the major compounds of homoisoflavonoids in *O. japonicus* root. Therefore, this study identified the main homoisoflavonoids in the extract of *O. japonicus* root by HPLC–DAD and LCMS/MS analyses, and evaluated the antioxidant activities of the extracts and two major homoisoflavonoid compounds (methylophiopogonanone A and methylphiopogonanone B).

## Materials and methods

*Materials, Chemicals and Reagents*

The roots of *O. japonicus* were collected from Mian Yang, Sichuan Province, China, in March 2012. Rutin, 2,2-diphenyl-1-picrylhydrazyl (DPPH), 2,4,6-tri(2-pyridyl)-s-triazine (TPTZ), 2,2-azi-nobis (3-ethylbenzothiazoline-6-sulfonic acid) (ABTS), 6-hydroxy-2,5,7,8-tetramethylchroman-2-carb- oxylic acid (trolox), and neocuproine were purchased from Sigma-Aldrich (St. Louis, MO, USA). Methylophiopogonanone A (MOPA) and methylophiopogonanone B (MOPB) were purchased from Pureone Biotechnology Co., Ltd. (Shanghai, China). Chloroform, methanol, and ethanol used for extraction were of analytical grade and purchased from XiLong Chemical Co., Lid. (Sichuan, China). Methanol, acetonitrile, and formic acid utilized for HPLC analysis were of HPLC-grade and purchased from Tedia Co., Inc. (Fairfield, USA).

*Extraction*

The dried root powder of *O. japonicus* was extracted with chloroform/methanol (1:1, v/v), methanol and 70% ethanol (20 g powder/200 mL of each solvent) by heat reflux for 2 h. The powder was extracted three times with each solvent. The extracts of each solvent were combined and filtered, respectively. The filtrates were evaporated to dryness to obtain the chloroform/methanol extract (CME), methanol extract (ME) and 70% ethanol extract (EE). Each extract was dissolved in methanol before use.

*HPLC–DAD analysis of the extracts*

HPLC analysis was carried out with a Water HPLC system (Waters, Milford, MA, USA), equipped with a SunFire C18 (Waters, Milford, MA, USA) column (250 mm×4.6 mm, 5 μM) and a Waters 2996 photodiode array detector (DAD). The mobile phase consisted of (A) acetonitrile/methanol (9:1, v/v) and (B) 0.05% aqueous formic acid (v/v). The gradient was as follows: 0 min – 41% A; 20 min – 41% A; 50 min – 44% A; 90 min – 65% A; 120 min – 65% A. For homoisoflavonoids, the detection was conducted at 296 nm, and the on-line UV spectra were recorded in the range of 200–400 nm. The running temperature was 30 °C, the injection volume was 20 μL and the flow rate was set at 1.0 mL/min.

*LCMS/MS analysis of the chloroform/methanol extract*

Homoisoflavonoids in the chloroform/methanol extract (CME) of *O. japonicus* root were identified using a LTQ XL linear ion trap mass spectrometer (Thermo Fisher Scientific Inc., San Jose, CA, USA). A SunFire C18 (Waters, Milford, MA, USA) column (250 mm×4.6 mm, 5 μM) was used for the separation. The mobile phase consisted of (A) acetonitrile/methanol (9:1, v/v) and (B) 0.05% aqueous formic acid (v/v) at a flow rate of 1.0 mL/min. The gradient was as follows: 0 min – 41% A; 20 min – 41% A; 50 min – 44% A; 90 min – 65% A; 120 min – 65% A. The injection volume was 20 μL, the split ratio was 1:2 and the column temperature was 30 °C. The full scan mass spectra were recorded from *m/z* 200 to 400 by electrospray ionization in negative ion mode. The ionization conditions were as follows: capillary temperature, 320 °C; spray voltage, 4.50 kV; sheath gas (N_2_) pressure, 45 psi; auxiliary gas (N_2_) pressure, 10 psi; source CID, 10 V; collision energy, 35 V.

*Determination of total flavonoid content*

Total ﬂavonoid content was determined using a modified method described by Sun *et al.* (18). Briefly, 0.5 mL of the diluted extract was mixed with 0.2 mL of a 5% (w/v) NaNO_2_ solution. After 6 min, 0.2 mL of a 10% (w/v) AlCl_3_ solution was added and allowed to stand for 5 min before 2 mL of 1 M NaOH solution was added. The mixture was adjusted to 5 mL with methanol. The absorbance was measured at 510 nm. The total ﬂavonoid content was quantiﬁed as mg of rutin equivalents per g of extract (mg RE/g).

*DPPH assay*

DPPH radical scavenging assay was carried out according to the method of Sarikurkcu *et al.* (19) with slight modifications. Briefly, 0.5 mL of sample in methanol was added to 3 mL of a 0.06 mM methanol DPPH radical solution. The solution was incubated in the dark at room temperature for 30 min. The absorbance of the solution was measured at 517 nm. Trolox was used as the reference compound, and the results are expressed as μmol of trolox equivalents per g of sample (μmol TE/g).

*ABTS assay*

ABTS radical scavenging assay was performed as described by Re *et al*. (20) with some modifications. Potassium persulfate was added to a 7 mM ABTS^•+^ solution at final 2.45 mM concentration. The mixture was kept for 12–16 h in the dark at room temperature. The ABTS^•+^ solution was diluted with ethanol to achieve an absorbance value of 0.7 ± 0.02 at 734 nm before use. Then, 0.5 mL of diluted sample was mixed with 2 mL of diluted ABTS^•+^ solution. The absorbance of the mixture was measured at 734 nm after 5 min at room temperature. Trolox was used as the standard, and the results are expressed as μmol of trolox equivalents per g of sample (μmol TE/g).

*FRAP assay*

Ferric reducing antioxidant power (FRAP) was determined using a modified method reported by Benzie and Strain (210. FRAP reagent was freshly prepared by mixing 50 mL of 300 mM acetate buffer (pH 3.6), 5 mL of 10 mM TPTZ in 40 mM HCl, and 5 mL of 20 mM FeCl_3_ solution. The FRAP reagent was incubated at 37 °C. Then, 0.05 mL of diluted sample was added to 3 mL of FRAP reagent. The solution was incubated for 4 min at 37 °C. The absorbance of the solution was recorded at 593 nm. The FRAP absorbance was determined by calculating the difference in absorbance of the sample and the control. Trolox was used as the standard, and the results are expressed as μmol of trolox equivalents per g of sample (μmol TE/g).

*CUPRAC assay*

Cupric reducing antioxidant capacity was determined according to a modified method of Apak *et al.* (22). Brieﬂy, 0.5 mL of diluted sample was mixed with 1 mL of 1 M NH_4_Ac buffer (pH 7.0), 1 mL of 7.5 mM neocuproine solution, 1 mL of 10 mM CuCl_2_ solution and 0.6 mL of deionized water. The absorbance of the mixture was measured at 450 nm after 1 h incubation at room temperature. Trolox was used as the reference compound, and the results are expressed as μmol of trolox equivalents per g of sample (μmol TE/g).

*Statistical analysis*

All tests were carried out in triplicate, and the data were expressed as mean ± SD (standard deviation). Statistical analysis was carried out using SPSS 17.0 and Excel 2013. Differences with a p value of < 0.05 were regarded as significant.

## Results and discussion

*HPLC–DAD analysis of the extracts*

**Figure 1:**
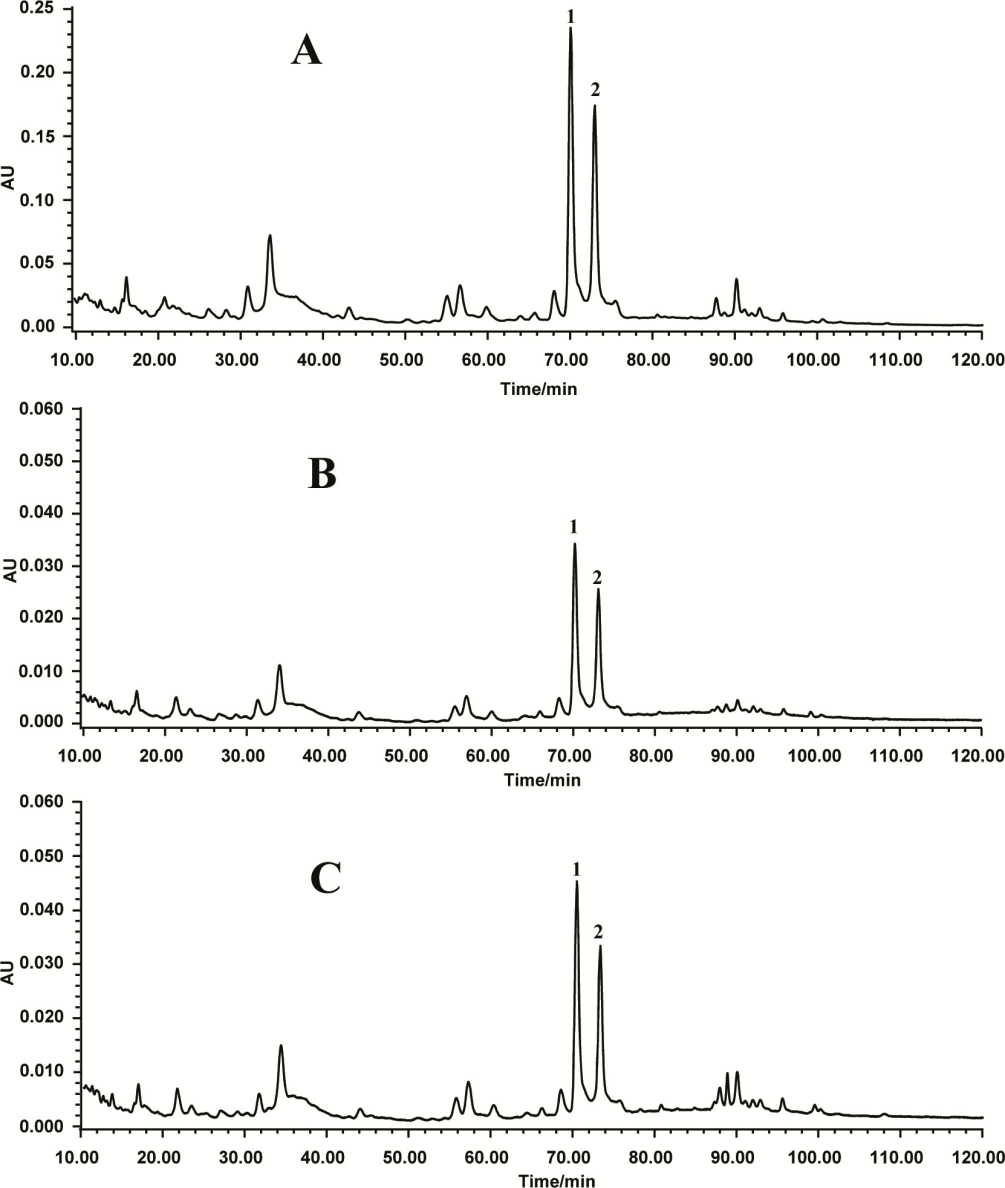
HPLC chromatogram of (A) chloroform/methanol extract, (B) methanol extract, and (C) 70% ethanol extract of *O. japonicus* root. Peak 1: methylophiopogonanone A; peak 2: methylophiopo- gonanone B

Figure 1. shows the HPLC–DAD chromatograms of CME, ME and EE of *O. japonicus* root recorded at 296 nm for homoisoflavonoids. Two major peaks (1 and 2) along with several other peaks with retention times around 12−36, 40−44, 35−36, 54−62, 63−69, and 86−98 min were showed in the chromatograms of three extracts. 

**Figure 2:**
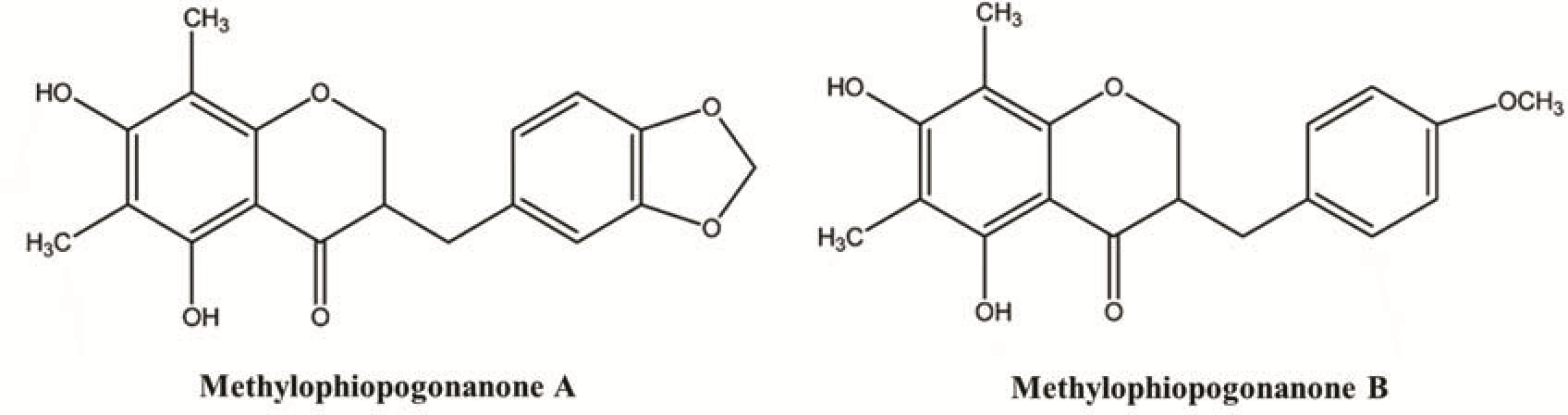
Chemical structures of methylophiopogonanone A and methylophiopogonanone B

Peak 1 and peak 2 were identified as methylophiopogonanone A (MOPA) and methylophiopogonanone B (MOPB) (Figure 2) by comparing their retention times with authentic standards, respectively. The relative distributions of MOPA, MOPB and other homoisoflavonoids in CME were much higher than in the other two extracts.

*Identification of homoisoflavonoids in CME*

**Figure 3:**
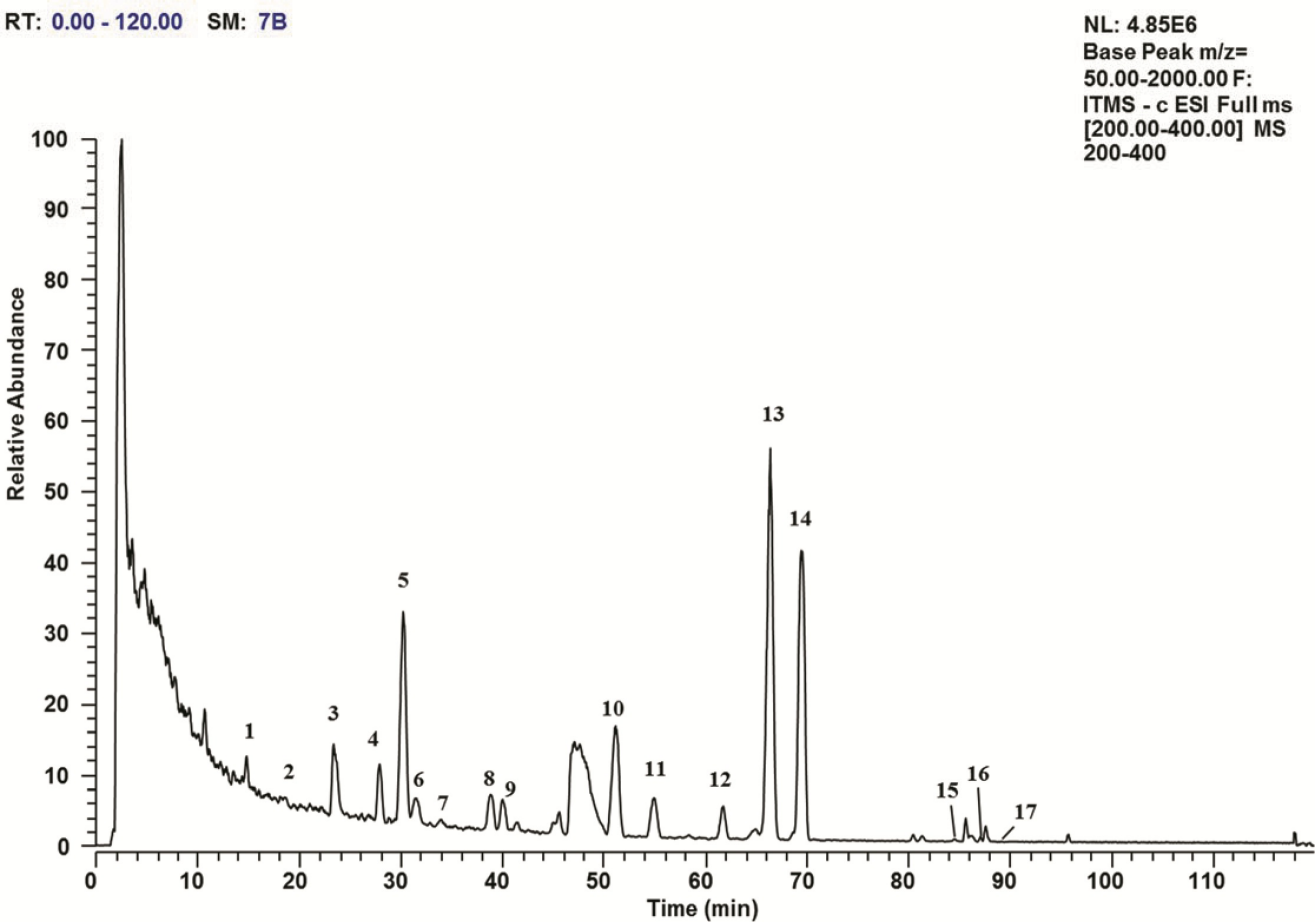
LCMS/MS total ion current profile in negative ion mode for the chloroform/methanol extract of *O. japonicus* root. The peak numbers show the homoisoflavonoids that are identified in Table 1

Homoisoflavonoids were identified by HPLC–DAD and LCMS/MS analyses and further confirmed by comparing their retention times, UV spectra, and MS/MS spectral data with those of authentic standards and information available in the literature. In the present study, CME of *O. japonicus* root was analyzed by LCMS/MS to obtain several peaks (Figure 3). A total of 17 homoisoflavonoids were characterized directly in CME of *O. japonicus* root without using additional purification steps (Table 1). Peak 13 and peak 14 were unambiguously identified as MOPA and MOPB by comparison of their retention times, and UV spectra and further confirmed using MS/MS spectral data of authentic standards, respectively. Other peaks were tentatively identified according to UV spectra, MS/MS spectrums and information reported in the literature.

Peak 1 (t_R_ = 14.81 min) and peak 3 (t_R_ = 23.80 min) which both gave a [M−H]^−^ ion at *m/z* 373 and had UV_λmax_ at 288 and 296 nm, respectively, were identified as 5,2›-dihydroxy-7,8,4›-trimethoxy- 6-methyl homoisoflavanone and 5,7,4›-trihydroxy-3›,5›-dimethoxy-6,8-dimethyl homoisoflavanone, respectively. Their MS/MS spectrums gave base peaks at *m/z* 183 and 207, respectively. Peak 2 (t_R_ = 19.45 min), peak 12 (t_R_ = 61.78 min) and peak 17 (t_R_ = 89.68 min) gave a same [M−H]^−^ ion at *m/z* at 339. Their MS/MS spectrums yielded prominent ions at 324, 311 and 311, respectively. Although peak 12 and peak 17 yielded similar MS/MS spectra, they had different UV_λmax_. Peak 12 and peak 17 had UV_λmax _at 265 and 274 nm, respectively. Therefore, peak 2, peak 12 and peak 17 were identified as 5-hydroxy-7,4›-dimethoxy-6,8-dimethyl homoisoflavone, methylophiopogonone A and 6-aldehydo- isoophiopogonone B by comparison of their mass spectrums with those reported by Lin *et al*. (23), respectively.

The mass spectrums of peak 5 (t_R_ = 30.34 min), peak 8 (t_R_ = 38.08 min) and peak 10 (t_R_ = 51.31 min) matched very well with that of ophiopogonanone E, 5-hydroxy-7-methoxy-3›,4›-methylenedioxy- 6,8-dimethyl homoisoflavanone and ophiopogonanone A, respectively (10). Peak 5 and peak 8 gave [M−H]^−^ ions at *m/z* 359 and 355, respectively, and both had UV_λmax_ at 296 nm. Peak 10 displayed [M−H]^− ^ion at *m/z* 327 and had UV_λmax_ at 294 nm.

**Table 1:** Massspectrometric data and identification of homoisoflavonoids in the chloroform/methanol extract (CME) of *O. japonicus* root

**Peak no.**	**t**_R _ **(min)**	**[M** **–** **H]** ^−^ ***m/z***	**MS/MS ** ***m/z *** **(relative intensity, %)**	**Compound**
1	14.81	373	358(18), 183(100), 168(28), 153(24)	5,2'-Dihydroxy-7,8,4'-trimethoxy-6-methyl homoisoflavanone a
2	19.45	339	324(100), 296(7)	5-Hydroxy-7,4'-dimethoxy-6,8-dimethyl homoisoflavone a
3	23.80	373	207(100)	5,7,4'-Trihydroxy-3',5'-dimethoxy-6,8-dimethyl homoisoflavanone a
4	27.87	343	207(100)	5,7,2'-Trihydroxy-4'-methoxy-6,8-dimethyl homoisoflavanone a
5	30.34	359	344(100), 223(12), 169(94), 154(47)	Ophiopogonanone E a
6	31.51	357	339(41), 222(6), 207(12), 153(100)	5,7-Dihydroxy-8-methoxy-3',4'-methylene-dioxy-6-methyl homoisoflavanone a
7	34.00	343	325(100), 207(38), 153(69)	5,7-Dihydroxy-8,4'-dimethoxy-6-methyl homoisoflavanone a
8	38.08	355	340(54), 205(100)	5-Hydroxy-7-methoxy-3',4'-methylenedioxy-6,8-dimethyl homoisoflavanone a
9	39.97	355	327(32), 218(38), 205(100)	5,7,2'-Trihydroxy-3',4'-methylenedioxy-6,8-dimethyl homoisoflavone a
10	51.31	327	205(28), 192(100), 164(46)	Ophiopogonanone A a
11	54.95	313	192(100), 164(18)	5,7-Dihydroxy-4'-methoxy-6-methyl homoisoflavanone a
12	61.78	339	311(100), 218(18), 179(10)	Methylophiopogonone A a
13	66.47	341	206(100), 178(52), 150(3)	Methylophiopogonanone A b
14	69.48	327	206(100), 178(25)	Methylophiopogonanone B b
15	84.57	353	325(100), 297(4)	6-Aldehydo-isoophiopogonone A a
16	87.12	355	337(23), 327(100), 307(60), 193(32)	6-Formyl-isoophiopogonanone A a
17	89.68	339	324(27), 311(100), 296(19)	6-Aldehydo-isoophiopogonone B a

a Identification based on HPLC–DAD analysis and MS/MS spectral data.

b Identification based on the authentic standard.

**Table 2:** Extraction yield and total flavonoid contentof the extracts of *O. japonicus* root

**Sample**	**Extraction yield (w/w, %)**	**Total flavonoid content** **(mg RE/g)**
CME	3.89 ± 0.15	16.50 ± 0.38
ME	26.42 ± 1.39	3.76 ± 0.16
EE	31.90 ± 1.42	2.62 ± 0.06

Values are expressed as mean ± SD of three determinations.

**Table 3:** Antioxidant activity of the extracts, MOPA, and MOPB of *O. japonicus* root determined by the DPPH, ABTS, FRAP and CUPRAC methods

**Sample**	**DPPH** **(μmol TE/g)**	**ABTS** **(μmol TE/g)**	**FRAP** **(μmol TE/g)**	**CUPRAC** **(μmol TE/g)**
CME	30.96 ± 0.26 c	45.54 ± 0.24 c	38.95 ± 0.59 b	132.64 ± 0.84 d
ME	9.38 ± 0.04 b	11.45 ± 0.42 b	8.22 ± 0.15 a	37.60 ± 0.76 b
EE	7.22 ± 0.04 a	9.39 ± 0.26 a	7.65 ± 0.20 a	22.66 ± 0.54 a
MOPA	31.56 ± 0.30 c	55.59 ± 1.30 d	225.03 ± 0.91 c	82.17 ± 0.79 c
MOPB	136.10 ± 0.94 d	163.90 ± 0.50 e	345.12 ± 0.64 d	217.00 ± 0.75 e

Values are expressed as mean ± SD of three determinations.

Different letters (a–e) in the same column indicate significant differences at p < 0.05.

MOPA: methylophiopogonanone A; MOPB: methylophiopogonanone B.

Peak 4 (t_R_ = 27.87 min) and peak 7 (t_R_ = 34.00 min) both gave a [M−H]^−^ ion at *m/z* 343 and had UV_λmax_ at 296 and 292 nm, respectively. Their MS/MS spectrums gave base peaks at *m/z* 207 and 325, respectively. Peak 6 (t_R_ = 31.51 min) and peak 9 (t_R_ = 39.97 min) gave [M−H]^−^ ions at *m/z* 357 and 355, and had UV_λmax_ at 296 and 265 nm, respectively. Their MS/MS spectrums gave base peaks at *m/z* 153 and 205, respectively. According to the MS/MS spectrums information reported by Lin *et al*. (23), peak 4, peak 6, peak 7 and peak 9 were identified as 5,7,2›-trihydroxy-4›-methoxy-6,8-dimethyl homoisoflavanone, 5,7-dihydroxy-8-methoxy-3›,4›-methylene-dioxy-6-methyl homoisoflavanone, 5,7- dihydroxy-8,4›-dimethoxy-6-methyl homoisoflavanone and 5,7,2›-trihydroxy-3›,4›-methylenedioxy-6,8 -dimethyl homoisoflavone, respectively.

Peak 11 (t_R_ = 54.95 min), peak 15 (t_R_ = 84.57 min) and peak 16 (t_R_ = 87.12 min) displayed [M−H]^− ^ion at *m/z* 313, 353 and 355, and had UV_λmax_ at 294, 274 and 274 nm, respectively. Their MS/MS spectrums gave base peaks at *m/z* 192, 325 and 327, respectively. The results are consistent with those previously reported (5, 9). Therefore, peak 11, peak 15 and peak 16 were identified as 5,7-dihydroxy-4› -methoxy-6-methyl homoisoflavanone, 6-aldehydo-isoophiopogonone A and 6-formyl-isoophiopogon- anone A, respectively.

*Total flavonoid content and antioxidant activities*

The total flavonoid content and extraction yield of CME, ME and EE of *O. japonicus* root are presented in Table 2. Among the extracts, the yield of CME was the lowest (3.89 ± 0.15%, w/w). However, the total flavonoid content of CME (16.50 ± 0.38 mg RE/g) was significantly higher than that of the other two extracts. It was in agreement with the results obtained by HPLC−DAD analysis in this study. The results indicated that most of the homoisoflavonoids in *O. japonicus* root were extracted into CME.

The antioxidant activities of three extracts (CME, ME and EE) and two homoisoflavonoid compounds (MOPA and MOPB) of *O. japonicus* root have been evaluated by the DPPH, ABTS, FRAP and CUPRAC assays. As shown in Table 3. the antioxidant activities decreased in the order of MOPB > MOPA > CME > ME > EE, according to the DPPH, ABTS and FRAP assays, with the exception of the rank order of MOPA and CME in the CUPRAC assay. Among three extracts, CME of *O. japonicus* root exhibited remarkable higher values of DPPH, ABTS, FRAP and CUPRAC (30.96 ± 0.26 μmol TE/g, 45.54 ± 0.24 μmol TE/g, 38.95 ± 0.59 μmol TE/g, and 132.64 ± 0.84 μmol TE/g, respectively) than that of ME and EE. The results of antioxidant abilities of the extracts matched with their total flavonoid contents. CME of *O. japonicus* root was proved to be abundant in homoisoflavonoids in this study. The two major homoisoflavonoid compounds (MOPA and MOPB) in the extracts showed strong antioxidant abilities. MOPB exhibited the strongest antioxidant activity of all test samples. The results demonstrated that the antioxidant activity of *O. japonicus* root is well correlated with the content of its homoisoflavonoid compounds.

## Conclusion

In this study, 17 homoisoflavonoid compounds in the *O. japonicus* root extract were identified. MOPA and MOPB were the two major compounds of homoisoflavonoids in the extracts. Our results showed that the root extract which contains substantial amounts of homoisoflavonoids exhibited significantly stronger antioxidant activities. Our studies might contribute to understand the biological activity and provide scientific support for the further investigation of *O. japonicus* root as a valuable source of raw material for drug application.
